# Characteristics and outcomes of pediatric extracorporeal cardiopulmonary resuscitation (E-CPR): a nationwide retrospective analysis from the French pediatric ECMO Network

**DOI:** 10.1016/j.aicoj.2026.100126

**Published:** 2026-07-25

**Authors:** R. Bernardon, N.C. Bulescu, T. Blache, C. Pavy, M. Grimaud, J. Rambaud, E. Langouet, A. Clairaz, S. Delangue, L. Berthomieu, S. Denante, N. Joram, A. Chenouard, A. Gaultier, P. Bourgoin, French Pediatric ECMO Network, Xavier Alacoque, Xavier Berreta Piccoli, Alice Bordessoule, Yvonnick Boué, César Brunetti, Ramy Charbel, Charlie Demelo, Aline Desrumeaux, Virginie Fouilloux, Roland Henaine, Julien Jegard, Pierre-Louis Leger, Yael Levy, Vanessa Lopez, Philippe Mauriat, Nicolas Roullet Renoleau, Ariane Willems

**Affiliations:** a Anesthesiology Department, CHU Nantes, F-44000 Nantes, France; b Department of Cardiac Surgery, CHU Lyon Hospices Civils, F-69029 Bron, France; c Department of Cardiac Surgery, Hopital Necker Enfants Malades AP-HP, F-75015 Paris, France; d Department of Pediatric Intensive Care, Hopital Necker Enfants Malades AP-HP, F-75015 Paris, France; e Department of Pediatric Intensive Care, Hopital Trousseau AP-HP, F-75012 Paris, France; f Department of Anesthesiology, Bordeaux University Hospital, F-33000, Bordeaux, France; g Department of Pediatric Cardiac Intensive Care, Hopital Marie Lannelongue, F-92350 Le Plessis Robinson, France; h Department of Pediatric Intensive Care, Hopital Jeanne de Flandre, F-59000 Lille, France; i Department of Pediatric Intensive Care, Hopital Toulouse, F-31300 Toulouse, France; j Department of Pediatric Intensive Care, APHM La Timone, F-13000 Marseille, France; k Department of Pediatric Intensive Care, Nantes University Hospital, CIC-FEA, F-44000 Nantes, France; l Department of Biostatistics, Nantes University Hospital, F-44000 Nantes, France

**Keywords:** Cardiac arrest, Extracorporeal membrane oxygenation, Pediatric intensive care

## Abstract

**Objective:**

To report the incidence and outcomes of E-CPR for intra or extra-hospital pediatric refractory cardiac arrest (CA) within the French national pediatric ECMO (Extra Corporeal Membrane Oxygenation) network.

**Methods:**

Retrospective study. Children (0–18 years) who underwent E-CPR for intra or extra-hospital cardiac arrest over a 5-year period (2019–2023) in one of the centers belonging to the network were included for analysis.

**Results:**

A total of 164 patients were included for analysis. Centers reported a median of 6 cases (range: 0–21). Most patients received E-CPR for intra-hospital CA (150, 91.5%), with cardiac origin suspected in 134 (84.3%) patients. Preexistent cardiac disease was reported in 94 patients (57.3%). Survival rate at PICU discharge, hospital discharge and after one-year was 29.9%, 29.3% and 27.4% respectively. We identified male gender (aOR 2.74, 95%CI 1.22 to 6.14, p = 0.014) and higher serum lactate 24 h after CA (aOR 1.11 per mol/L, 95%CI 1.03–1.18, p = 0.003) as independent factors associated with poor prognosis whereas femoral cannulation site (aOR 0.18, 95%CI 0.06 to 0.52, p = 0.002) was associated with favorable outcome.

**Conclusion:**

E-CPR is mostly performed in children with heart disease, with limited use in out-of-hospital settings. Efforts should be made to identify patients in need of ECMO before CA, as the outcome after E-CPR remains poor.

## Background

Survival rates after cardiac arrest (CA) in children are mitigated. Survival rates after out-of-hospital CA (OHCA) more or less20% [1,2], while it may reach 40% after in-hospital CA (IHCA) [3], with half of these children presenting significant cognitive impairment and impaired daily functioning one year after CA [4]. Low flow (LF) duration, defined as the duration of cardiopulmonary resuscitation before return of spontaneous circulation (ROSC) exceeding 20 min, is one of the major prognostic factors, alongside with the location of the cardiac arrest (IHCA/ OHCA), the type of cardiac arrest (shockable rhythm or asystole), and patient's condition (post-cardiotomy, known heart disease) [5,6]. E-CPR (Extracorporeal Cardio-Pulmonary Resuscitation) is therefore a way to reduce the duration of LF in order to improve the prognosis of patients in CA. The definition of E-CPR adopted by ELSO (Extracorporeal Life Support Organization) [7] is the deployment of ECMO (Extra Corporeal Membrane Oxygenation) after CA when conventional cardiopulmonary resuscitation (C-CPR) fails to achieve ROSC or when ROSC is not sustained beyond 20 min. The first E-CPR series in children was reported after congenital cardiac surgery with encouraging results in 1992 [8]; Several pediatric studies have since provided arguments in favor of E-CPR as compared to C-CPR in the context of IHCA [9,10]. The 2021 ILCOR (International Liaison Committee on Resuscitation) recommendations [11] as well as 2025 ERC guidelines [12] mention the use of E-CPR in selected children with IHCA, particularly during heart surgery or in the context of intractable cardiac arrhythmia. E-CPR in children remained a marginal practice with 11 cases per year reported in 2017 within the French Pediatric ECMO Network [13].

The main objective of this multicenter retrospective study conducted in France (mainland and overseas territories) was to describe the characteristics of children receiving ECMO for E-CPR as well as to report outcomes after E-CPR.

## Material and methods

### Study design

Retrospective multicenter study conducted by the French Pediatric ECMO Network. The study was declared at Nantes, University hospital, and an Institutional Review Board (Nantes Health Ethics Group) approved the study (PROMOPED-study: 24-41-03-270; 03/27/2024) and waived consent for the collection of existing de-identified data, in accordance with the ethical standards of the responsible committee on human experimentation and with the Helsinki Declaration of 1975.

### Patients

All children <18 years old receiving E-CPR for IHCA or OHCA during a 5-years period, from January 1st, 2019, to December 31st, 2023, within one of the centers of the network (the 18 pediatric ECMO centers contributing to the network are described in supplementary materials Table S1). Center characteristics, along with patient demographics and brief medical histories, were documented. Circumstances surrounding CA were detailed, including location, witness presence, initial cardiac rhythm, and suspected etiology. No flow (NF) and low flow (LF) duration were the time from CA to CPR and the time from initiation of conventional CPR and to initiation of E-CPR. Clinical and laboratory assessments were performed at the time of cannulation (using the closest value within six hours prior) and again after 24 h (using the nearest values obtained between 18–30 h post-cannulation). ECMO parameters such as cannulation site, 24-h flow rate, and complications—defined according to ELSO criteria—were also recorded. Outcomes assessed included survival at 24 h, at the time of ECMO weaning, at PICU discharge, at hospital discharge, and one-year survival. Neurological status at Pediatric Intensive Care Unit (PICU) discharge was evaluated using the Pediatric Cerebral Performance Category (PCPC) [14].

### Statistical analysis

The initial phase of the statistical analysis involved summarizing the demographic data for the entire population, followed by a comparative assessment of the characteristics between the survivor and non-survivor groups. Quantitative data were expressed as the mean (standard deviation), and qualitative data were expressed as numbers and percentages relative to the population of the group concerned. The correlation between two continuous variables was tested using a Pearson correlation test. Bivariate analyses between outcomes and patient characteristics were performed using Student's t-tests for continuous variables or Chi-square or Fisher's t-test (for small sample sizes) for categorical variables. On an exploratory basis, we sought to determine the variables associated with survival at hospital discharge using multivariate mixed logistic regression adjusted for center as random effect. Candidate variables for constructing multivariate models were those with a significance threshold <0.1, or those clinically relevant (age, ECMO volume per center). In order to retain in the final multivariate model only the statistically significant or clinically relevant variables, final multivariate models were constructed using stepwise model selection based on the Bayesian Information Criterion (BIC). Factors associated with poor prognosis were assessed at the time of cannulation and after 24 h after exclusion of children died during the first 24 h of support. Factors associated with poor prognosis were assessed at the time of cannulation and after 24 h after exclusion of children died during the first 24 h of support. A two-tailed p-value <0.05 was used as the threshold for statistical significance. Statistical analysis was performed using R 4.4.1.

## Results

### Participating centers

All pediatric ECMO centers of the network (n = 18) participated in this study. A total of 1,105 ECMO cannulations were reported during the study period, including 164 (14.8%) E-CPR procedures from 17 centers (one center did not perform any E-CPR). The number of E-CPR cases per center over five years ranged from 1 to 21. In two centers, E-CPR was marginal, with one case reported during the entire period. Six centers reported an average of ≤1 E-CPR case per year. An upward trend in E-CPR utilization was noted, with 57 cases (34.8%) occurring in the first half of the study period and 107 cases (65.2%) in the latter half. A majority of E-CPR occurred in centers performing routine pediatric cardiac surgery (n = 147, 89.6%). E-CPR was initiated in center declaring more than 20 ECMO per year in 68 (41.5%). Description of ECMO centers, as well as the number of E-CPR reported are described in supplemental Table S1.

### Characteristics of patients, circumstances of CA and ECMO characteristics

Patient’s characteristics and cardiac arrest circumstances are summarized in Table 1. Twenty-six patients were neonates (15.9%) with 5 born preterm. Most patients had pre-existing medical condition before CA, including 84 (51.2%) with a history of congenital heart disease. In 42 (25.6%) patients, no medical history was reported before CA. Accidental drowning was reported in 3 (1.8%) patients, associated with severe hypothermia in two patients (26 °C and 30 °C respectively). The vast majority of CA (150, 91.5%) occurred in hospital settings. Of them, 108 (72.0%) occurred in the PICU, 23 (15.3%) in the Operating Room (OR), 12 (8.0%) in the pediatric ward. Seven CA (4.7%) occured in a non-ECMO center, and CA location was not reported in 14 cases (8.5%). There were 14 E-CPR for OHCA, with 12 out of 14 (85.7%) resulting in death before hospital discharge. One survivor, a 15 year old child had experienced shockable cardiac arrest, LF duration 66 min, leading to the diagnosis of hypertrophic cardiomyopathy, and was discharge from hospital with severe disability. The second survivor was a 12 year old child with known Brugada syndrome, experiencing shockable cardiac arrest at school, transported to the ECMO center after ROSC, and presenting repeated CA at the time of hospital admission with a cumulated LF duration of 30 min, and favorable neurological outcome. Most of CA were witnessed (159, 96.9%), and a NF duration was reported in 7 patients only (minimal NF duration 1 min, maximal 30 min), with 2 survivors experiencing respectively NF duration of 1 and 5 min. LF duration was specified in 159/164 (97%) of the patients. Mean LF duration is reported in Table 1. LF duration was not specified in one survivor, presenting a sequence of multiple CA with ROSC lasting over 20 min, for a total duration of 132 min. Otherwise, the maximal LF duration was 81 min in survivors and 220 min in non-survivors. No survival was noted for LF duration >80 min. The mean LF duration within the cohort was 49.9 ± 33.5 min.

**Table 1:** Patient’s characteristics and circumstances of cardiac arrest according to the survival at hospital discharge.

	Overall 164 (100%)	Non-survivors 116 (70,7%)	Survivors 48 (29,3%)	*Sig.*
Patients characteristics				
Age (years), mean (SD) (n = 164)	3.86 (4.53)	3.53 (4.33)	4.66 (4.9)	*0.20*
Neonate (n = 164)	26 (15.8%)	20 (17.2%)	6 (12.5%)	*0.32*
Weight (kg), mean (SD) (n = 164)	15.9 (15.8)	14.7 (15.5)	18.6 (16.4)	*0.16*
Sexe (n = 164)				*0.018*
Female (%)	66 (40.7%)	41 (36.0%)	25 (52.1%)	
Male (%)	96 (59.3%)	73 (64.0%)	23 (47.9%)	
Not reported	2	2	0	
Comorbidities (n = 164)				
Genetic or syndromic association	20 (12.2%)	13 (11.2%)	7 (14.6%)	*0.55*
Aquired or congenital heart disease	84 (51.2%)	23 (47.9%)	61 (52.6%)	*0.67*
Oncology, hematology	5 (3.0%)	3 (2.6%)	2 (4.2%)	*0.68*
No comorbity	42 (25.6%)	27 (23.3%)	15 (31.2%)	*0.38*
Cardiac arrest characteristics				
Bystander (Y/N) (n = 164)				*0.99*
Yes	159 (96.95%)	111 (95.69%)	48 (100%)	
No	5 (3.05%)	5 (4.31%)	0 (0%)	
CA location (n = 164)				*0.29*
Out of hospital	14 (8.5%)	12 (10.3%)	2 (4.2%)	
In hospital	150 (91.5%)	104 (89.7%)	46 (95.8%)	
First rhythm monitored (n = 164)				*<0.001*
Shockable (VF/VT)	36 (21.9%)	14 (12.1%)	22 (45.8%)	
Non-shockable (PEA/ Asystole)	127 (77.4%)	101 (87.1%)	26 (54.2%)	
Unknown	1 (0.6%)	1 (0.9%)	0 (0%)	
Suspected causes of CA (n = 164)				*0.01*
Cardiac	40 (24.4%)	23 (19.8%)	17 (35.4%)	
Cardiac -post-cardiotomy)	58 (35.4%)	41 (35.3%)	17 (35.4%)	
Cardiac (known cardiopathy)	36 (22.0%)	24 (20.6%)	12 (25.0%)	
Respiratory	15 (9.1%)	14 (12.1%)	1 (2.1%)	
Drowning	3 (1.8%)	3 (2.6%)	0 (0%)	
Unknown	12 (7.3%)	11 (9.5%)	1 (2.1%)	
Low flow (min), mean (SD) (n = 159)	49.9 (33.5)	55.2 (35.4)	37.4 (24.6)	*0.004*

CA = Cardiac Arrest; ECMO = Extra Corporeal Membrane Oxygenation; VF: ventricular fibrillation, VT: pulseless ventricular tachycardia.

All patients received veno-arterial ECMO support. Femoral cannulation was reserved for older patients; the youngest pateint supported through femoral access was 927 days old (approximately 3 years), weighing 14.6 kg. The mortality across time period, 36/57 (63.2%) and 80/107 (74.2%) respectively for the first and second half of the inclusion period was not different (*p = 0.168),* as it was for center ECMO volume, [mortality: 24/28 (85.7%); 49/68 (72.1%); and 43/68 (63.2%) respectively for center declaring annual ECMO volume less than 10, 10–20, or up to 20 ECMO cases per year, *p = 0.08*). Characteristics of ECMO and assessment at the time of cannulation as well as 24-hs assessment are reported in Table 2. Overall, patients were supported with ECMO during a mean of 4.7 ± 5.4 days. Survivors spent more time on ECMO than non-survivors (6.6 ± 4.0 days vs 3.7 ± 5.7 days, p < 0.001) due to early deaths in the latter group. During the course of ECMO, mechanical complications were noted in 30 (18.3%) patients, bleeding complications in 57 (34.8%) patients. A neurological complication was noted during the course of ECMO in most patients (102; 62.2%), including brain death in 45 patients (44.1%). In survivors, an acute neurological event (clinically or ElectroEncephaloGram (EEG) determinated seizure, Central Nervous System (CNS) infarction or CNS hemorrhage) was noted in 19 (39.6%). Lower limb ischemia was reported in 8 patients (4.9%), with the need of amputation in 2 survivors.

**Table 2:** Characteristics of ECMO support and biological values at cannulation and 24 h assessment according to survival at hospital discharge. Results are presented as mean (SD) or n (%).

	Overall 164 (100%)	Non-survivors 116 (70.7%)	Survivors 48 (29.3%)	*Sig.*
Characteristics at cannulation				
Cannulation site (n = 160)				*0.017*
Neck	62 (38.7%)	50 (44.6%)	12 (25.0%)	
Central	58 (36.2%)	39 (34.8%)	19 (39.6%)	
Femoral	37 (23.1%)	20 (17.9%)	17 (35.4%)	
Other	3 (1.9%)	3 (2.7%)	0 (0%)	
Location (n = 162)				
ECMO center	145 (89.5%)	105 (91.3%)	40 (85.1%)	*0.377*
Remote	17 (10.5%)	10 (8.7%)	7 (14.9%)	
Serum lactate (mmol/L) (n = 153)	13.54 (6.30)	14.31 (6.31)	11.64 (5.94)	*0.021*
pH(n = 153)	7.03 (0.23)	6.99 (0.23)	7.12 (0.21)	*0.006*
Serum bicarbonate (mmol/l)(n = 139)	14.25 (7.22)	13.21 (6.67)	16.75 (7.93)	*0.010*
PaO_2_ (mmHg) (n = 133)	170.18 (149.38)	162.54 (149.09)	189.99 (150.36)	*0.388*
PaCO_2_ (mmHg)(n = 140)	54.09 (24.29)	55.82 (26.44)	49.76 (17.37)	*0.541*
PT (%) (n = 114)	49.88 (21.63)	49.08 (21.37)	51.61 (22.38)	*0.364*
Platelet count (Giga/L)(n = 131)	171.80 (131.47)	165.04 (140.14)	188.34 (107.26)	*0.205*
24-h assessment (n = 138 patients assessed)				
	Overall	Non-survivors	Survivors	*Sig.*
Lactate (mmol/L) (n = 113)	3.43 (3.18)	4.62 (3.62)	1.76 (1.14)	*< 0.001*
pH H24 (n = 113)	7.37 (0.10)	7.35 (0.12)	7.39 (0.07)	*0.010*
Bicarbonates at H24 (mmol/L) (n = 104)	25.81 (5.73)	26.02 (6.18)	25.48 (5.00)	*0.307*
PaO_2_ H24 (mmHg)(n = 103)	135.65 (103.06)	136.72 (114.26)	133.97 (83.78)	*0.979*
PaCO_2_ H24 (mmHg) (n = 103)	44.60 (8.36)	45.96 (9.18)	42.45 (6.42)	*0.219*

ECMO: extracorporeal membrane oxygenation; PT: prothrombin time (expressed as a ratio of value observed in controls).

### Outcomes

**Fig. 1:**
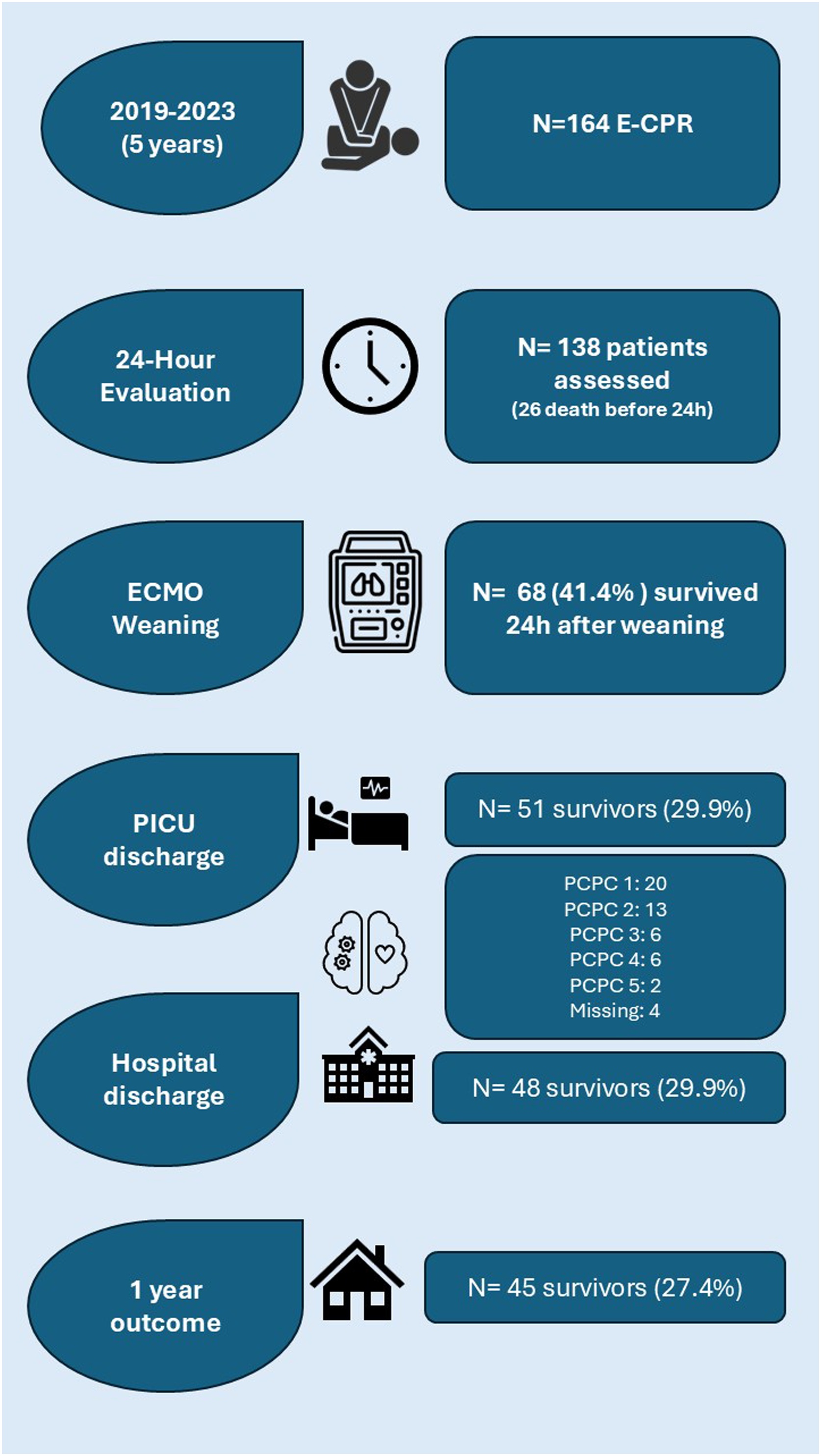
Outcomes of patients receiving E-CPR during.

Survival rates for specified time points were available in all patients (Fig. 1**)**. Survival rate at hospital discharge was 48/164 (29.7%). Among 51 survivors at PICU discharge, Neurological status was quoted PCPC 1–3 (no disability, mild or moderate disability) in 39 (76.5%) of the patients, PCPC 4–5 (severe disability, coma or vegetative state) in 8 (15.7%), and not reported in 4 (8.3%) of the patients, with all of them noted alive after one year.

### Factors associated with death at hospital discharge

**Fig. 2:**
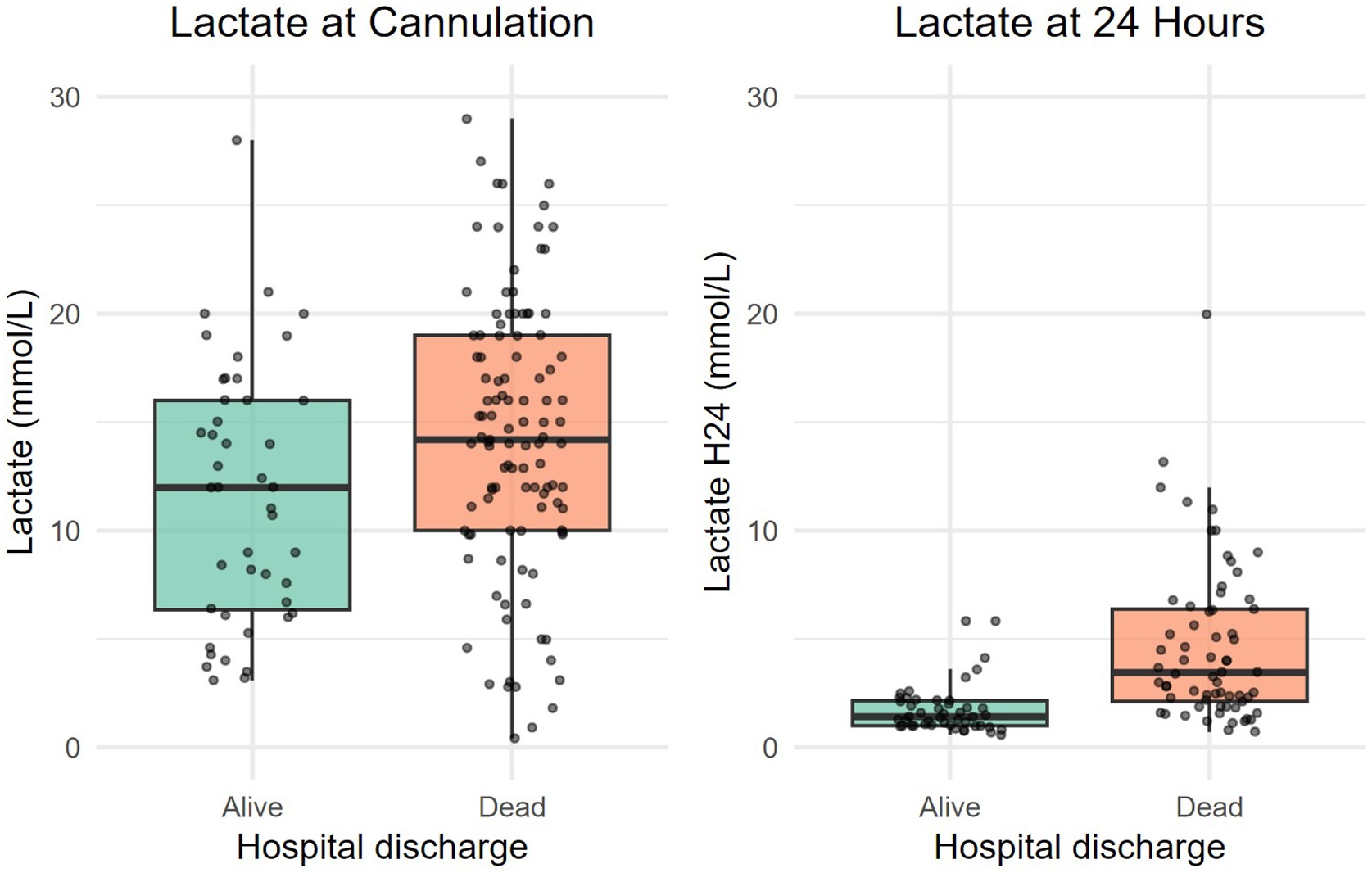
Serum lactate level at the time of E-CPR and during 24-hour assessment, according to the status at hospital discharge.

Results of univariate analysis are shown in Table 1, Table 2. Fig. 2 depicts the distribution of serum lactate level at the time of cannulation and after 24 -hs assessment. There was no correlation between serum lactate level at cannulation and after 24 -hs (Pearson r = 0.048; p = 0.59). In survivors, the maximal serum lactate level at 24 h was 5.8 mmol/L. The predictive performance of serum lactate level was poor at the time of cannulation, as compared to values recorded after 24 h (AUC of the ROC curve 0.62, 95%CI: 0.52−0.72 versus 0.84, 95% CI: 0.77−0.91, respectively). In a multivariate analyses included variables available at the time of cannulation only and variables available after 24 -hs assessment separately, we identified male gender (aOR 2.74, 95%CI 1.22–6.14, p = 0.014) and higher serum lactate 24 h after CA (aOR 1.11 per mol/L, 95%CI 1.03–1.18, p = 0.003) as independent factors associated with poor prognosis whereas femoral cannulation site (aOR 0.18, 95%CI 0.06 to 0.52, p = 0.002) was associated with favorable outcome. Results are presented in Table 3. Corresponding receiver-operating characteristic curves at cannulation and after 24 h are presented in supplemental Figure S1.

**Table 3:** Multivariate analysis of predictors of death at hospital discharge, including factors recorded at the time of cannulation and after 24-h assessment.

	aOR	95%CI	*Sig.*
ECMO cannulation			
Gender			
Female	Ref		
Male	2.90	[1.21; 6.97]	*0.017*
First rhythm monitored			
Shockable	Ref		
Non-shockable	6.91	[2.44; 19.54]	*< 0.001*
Cannulation site			
Neck	Ref		
Central	0.25	[0.09; 0.74]	*0.012*
Femoral	0.23	[0.07; 0.75]	*0.014*
Serum lactate (per mmol/L)	1.10	[1.02; 1.18]	*0.010*
24-h assessment			
Gender			
Female	Ref		
Male	3.80	[1.26; 11.40]	*0.017*
First rhythm monitored			
Shockable	Ref		
Non-shockable	3.12	[0.95; 10.24]	*0.061*
Cannulation site			
Neck	Ref		
Central	0.30	[0.09; 1.02]	*0.054*
Femoral	0.08	[0.02; 0.43]	*0.003*
24 -h serum lactate (per mmol/L)	2.28	[1.48; 3.52]	*< 0.001*

aOR: adjusted Odds Ratio.

## Discussion

In this nationwide retrospective analysis conducted through the French Pediatric ECMO Network, we describe contemporary patterns of use, clinical characteristics, and outcomes associated with E-CPR in children over a five-year period in France. Our study, representing the largest French pediatric cohort to date, highlights the persistent challenges of managing refractory cardiac arrest in children and underscores prognostic factors. Three principal findings emerge. First, E-CPR remains predominantly an in-hospital rescue strategy for children with underlying cardiac conditions, with an increase in use during the second half of the study period. Second, overall survival remains modest at approximately 30%, but neurological outcomes among survivors are generally favorable, with most of survivors (39/51, 76.4%) exhibiting only mild to moderate disability at PICU discharge. Third, lactate concentration 24 h after cannulation is a strong and independent predictor of poor outcome, whereas LF duration—traditionally regarded as a key determinant in conventional CPR—did not remain significant in multivariate analysis when patient’s characteristics and early post-cannulation outcomes were considered.

E-CPR represented nearly 15% of all ECMO cannulations in participating centers, reflecting the increasing integration of extracorporeal rescue strategies into pediatric critical care. This proportion is notably higher than the roughly 10 cases per year previously reported in France [13], indicating a shift toward more systematic use of E-CPR, particularly in centers with established cardiac surgery programs. Nevertheless, utilization remained extremely heterogeneous across centers, with several institutions reporting only isolated cases. The observed variability may suggest that both local expertise and institutional culture play significant roles in the decision to initiate E-CPR, but the presence of local protocol for the initiation of E-CPR was not recorded in this study. Notably, considerable differences across centers regarding the initiation of E-CPR have been reported in adult populations [15], particularly in relation to ECMO eligibility for out-of-hospital cardiac arrest (OHCA). Another source of variability concerns the definition of E-CPR itself. While the mean and median LF) durations—representing conventional CPR time—in our study are consistent with values from the recent ELSO registry report, which cites a total CPR duration of 41 [26, 57] minutes [16], it is noteworthy that 12 patients in our cohort experienced LF durations of 10 min or less. This finding highlights a potential definitional bias, as there appears to be ambiguity between cases of pre-ECMO cardiac arrest occurring during cannulation and instances involving ECMO deployment for primary in-hospital cardiac arrest (IHCA).

More than 90% of arrests occurred in hospitals, mainly in highly monitored environments such as PICUs or operating rooms. Only 14 E-CPR for OHCA were recorded during the study period, reflecting well-recognized barriers to mobile ECMO deployment in the pediatric population, including logistical complexity, limited availability of trained transport teams, challenges related to cannulation in small children, as previously mentioned by Barbaro et al. [17], with OHCA consisting in 3% only of E-CPR in children. Furthermore, CA of unknown or non-cardiac origin are recognized to lead to poor outcomes, even in IHCA [16]. Outcomes reported for OHCA in France are notably poorer than those reported in a recent analysis of ELSO registry [18]. However, due to the limited number of patients with OHCA included, no conclusion may be drawn from the present study.

Despite early initiation of advanced life support and nearly universal witnessed arrest, overall survival within our network remained below 30%. Survival rates reported in the literature vary considerably. In a recent meta-analysis involving 1,348 children [19], survival rate was 46% overall, whereas the survival rate after E-CPR reported in the ELSO registry between 2009 and 2022 with 44% and 41% in the neonatal and pediatric population, respectively [20]. This discrepancy may be attributed to patient selection, with a relatively low proportion of patients receiving E-CPR after cardiac surgery (36.5%) in our cohort. It is indeed known that factors associated with higher survival after E-CPR in children include firstly the use of E-CPR in children with heart disease and especially in the perioperative period [4], where it is acknowledged that ECMO procedures for peri-procedural CA are the most effective. With the same idea, we noted limited volume of E-CPR cases in most network-affiliated centers. Only 8 out of 18 centers reported at least 10 E-CPR procedures during the study period. Both a post-surgical setting and higher E-CPR volume have been associated with improved outcomes [21].

Multivariate analysis identified elevated lactate levels 24 h after cannulation as independent predictors of mortality. The prognostic relevance of 24 -h lactate is physiologically plausible. It appears that 24-h lactate is not correlated with lactate at cannulation, and likely reflects the adequacy of extracorporeal support, tissue perfusion, and organ recovery. In the present study, none of the patients with persisting elevated lactate (> 6 mmol/L) after 24 h survived. While lactate at cannulation has traditionally been considered an indicator of preceding low-flow state and global hypoperfusion, its utility at the time of ECMO initiation appeared limited, with poor predictive value identified. From a clinical perspective, incorporating 24-h physiological parameters into prognostic models could assist teams in refining therapeutic goals, anticipating complications, and engaging in early discussions with families regarding prognosis. Moreover, centers might consider standardized protocols emphasizing early lactate clearance as a quality indicator for post-E-CPR care.

The initial monitored rhythm in pediatric cardiac arrest carries important prognostic value, with shockable rhythms (ventricular fibrillation or pulseless ventricular tachycardia) generally associated with higher survival rates and better neurological outcomes compared to non-shockable rhythms such as asystole or pulseless electrical activity. Although shockable rhythms are less common in children than in adults, their presence often reflects a more reversible etiology and a greater likelihood of response to timely defibrillation. Consequently, early rhythm identification and targeted management remain critical components of pediatric resuscitation strategies [22].

LF duration is a well-established determinant of outcome in conventional CPR strategies, and numerous studies have demonstrated the steep decline in survival associated with prolonged CPR. In our cohort, LF duration remained associated with mortality in univariate analysis but lost significance in multivariate modeling. Several explanations may account for this observation. First, ECMO support fundamentally modifies the relationship between LF duration and outcome. While prolonged CPR is unquestionably deleterious, successful E-CPR can restore perfusion even after extended periods of conventional CPR, making the absolute LF duration less informative once extracorporeal support is established. Indeed, survival was observed in our cohort even after LF durations approaching 80 min, consistent with the concept that E-CPR can, in selected cases, compensate for prolonged low-flow periods. Furthermore, LF duration is often imprecisely measured, especially in complex resuscitation scenarios involving recurrent episodes of ROSC. Our dataset included at least one case with multiple arrest sequences and intermittent ROSC, illustrating the difficulty in capturing a single reliable LF value.

Strengths and limitations. The main strength of this study lies in its nationwide scope and inclusion of all major pediatric ECMO centers in France, providing a realistic picture of E-CPR practice. The large sample size, spanning diverse institutions and patient profiles, enhances the external validity of our findings. Standardized data collection, adherence to ELSO-defined complications, and systematic reporting of neurological outcomes further strengthen the dataset.

Several limitations must be acknowledged. First, as a retrospective study, unmeasured confounding is inevitable. Decisions to initiate E-CPR were not standardized and likely varied across centers depending on expertise, resources, and clinical judgment. Second, although the cohort includes 164 patients, subgroup analyses remain underpowered, particularly for assessing rare complications or evaluating outcomes by age category or underlying pathology. Third, LF duration and NF duration may have been inconsistently reported, given the challenges inherent to documentation during high-intensity resuscitation efforts. Fourth, neurological outcomes were assessed only at PICU discharge, precluding long-term neurodevelopmental evaluation.A prospective cohort study would offer better identification of selection criteria and long term follow up, as it was implemented for OHCA [23].

## Conclusion

E-CPR in children is increasingly implemented within the French Pediatric ECMO Network, though its use remains concentrated in cardiac centers and primarily limited to in-hospital arrests. Despite improved access, outcomes after pediatric E-CPR remains poor, emphazing the need for standardized E-CPR pathways, promote earlier identification of children at risk of collapse—ideally before cardiac arrest occurs. Such efforts may help refine patient selection, optimize care, and ultimately improve survival for this highly vulnerable population.

## Author’s contribution

**BR:** data collection, analysis, manuscript writing**; BNC:** data collection; revised the manuscript; **BT**: data collection; revised the manuscript; **PC:** data collection; revised the manuscript; **GM**: data collection; revised the manuscript; **RJ**: data collection; revised the manuscript; **LE**: data collection; revised the manuscript; **CA**: data collection; revised the manuscript; **DS**: data collection; revised the manuscript; **BL**: data collection; revised the manuscript; **DS**: data collection; revised the manuscript; **JN**: data analysis, manuscript writing revised the manuscript; **CA**: data analysis, manuscript writing revised the manuscript; **GA**: Biostatistical analysis; **PB**: Study coordinator, analysis, manuscript writing.

## Consent for publication

Not applicable.

## Ethics approval and consent to participate

The study was declared at Nantes, University hospital, and an Institutional Review Board (Nantes Health Ethics Group) approved the study (PROMOPED-study: 24-41-03-270; 03/27/2024) and waived consent for the collection of existing de-identified data, in accordance with the ethical standards of the responsible committee on human experimentation and with the Helsinki Declaration of 1975.

## Funding

The present work was not funded.

## Availability of data and materials

Data are available on reasonable request

## Collaborators and Investigators from the French Pediatric ECMO Network

Xavier ALACOQUE (Toulouse), Redcap Administrator, Xavier BERRETA PICCOLI (Martinique); Alice BORDESSOULE (Genève), Yvonnick Boué (Mayotte), César BRUNETTI (Marseille), Ramy CHARBEL (Le Kremlin Bicêtre), Charlie DEMELO (Strasbourg), Aline DESRUMEAUX (Grenoble), Virginie FOUILLOUX (Marseille), Roland HENAINE (Lyon), Julien JEGARD (Nantes), Pierre-Louis LEGER (Paris), Yael LEVY (La Réunion), Vanessa LOPEZ (Paris), Philippe MAURIAT (Bordeaux), Nicolas ROULLET RENOLEAU (Tours), Ariane WILLEMS (Bruxelles).

## Data availability

The data that has been used is confidential.

Data will be made available on request.

## Declaration of competing interest

PB received funds from Medtronic© (consultant fees) and Eurosets© (travel reimbursement) for another works.
