# Supplementary material for: Characteristics and outcomes of pediatric extracorporeal cardiopulmonary resuscitation (E-CPR): a nationwide retrospective analysis from the French pediatric ECMO Network
Source: Ann Intensive Care. 2026 Jul 25;16:100126. doi: 10.1016/j.aicoj.2026.100126 (PMC13476720; doi:10.1016/j.aicoj.2026.100126)
Supplement: Supplementary file 1 [file mmc1.docx]

**Supplemental materials.**

**Supplemental figure S1:** Graphical representation of covariate selection using stepwise procedure, based on the Bayesian Information Criterion (BIC). In the Stepwise Regression process, predictors are added or removed based on specific criteria to improve the model’s accuracy and efficiency (*ie*, the lowest BIC value).

**Fig S1A:** covariates available at cannulation (lactate refers to the value reported with the closest time before cannulation).

**Fig S1B:** covariates available after 24h.

**Supplemental figure S2** : receiver-operating characteristic (ROC) curve for the prediction of death: Serum lactate level at the time of cannulation (figure S1.A), serum lactate level at 24 hours (figure S1.B), multivariate ROC curve with variables available at the time of cannulation: sex, first rhythm monitored, serum lactate level, and cannulation site (figure S1.C) and after 24 hours: sexe, first rhythm monitored, 24h serum lactate level, and cannulation site (figure S1.D).

Figure S1.A

AUC : 0.62

95%CI : 0.52-0.72

Figure S1.B

AUC : 0.83

95%CI : 0.77-0.91

Figure S1.C

AUC : 0.78

95%CI : 0.70-0.86

Figure S1.D

AUC : 0.89

95%CI : 0.83-0.95

**Supplemental table S1 : Participating centers and local investigators**

| **Name center** | **Unit description** | **Annual ECMO volume (declared)** | **ECPR (reported)** |
| --- | --- | --- | --- |
| CHU Trousseau (Paris) | Medical | > 20 | 4 |
| CHU Lyon | Surgical (cardiac) | > 20 | 15 |
| CHU Necker (Paris) | Medical | > 20 | 13 |
| CHU Marseille | Polyvalent | > 20 | 15 |
| CHU Nantes | Polyvalent | > 20 | 21 |
| CHU Necker (Paris) | Surgical (cardiac) | 10-20 | 19 |
| CCML Marie Lannelongue | Surgical (cardiac) | 10-20 | 21 |
| CHU Toulouse | Polyvalent | 10-20 | 16 |
| Kremlin Bicêtre | Medical | 0-10 | 3 |
| CHU Bordeaux | Surgical | 10-20 | 7 |
| CHU Martinique | Polyvalent | 0-10 | 4 |
| CHU Mayotte | Medical | 0-10 | 1 |
| CHU Réunion | Polyvalent | 0-10 | 4 |
| CHU Strasbourg | Polyvalent | 0-10 | 1 |
| CHU Tours | Polyvalent | 10-20 | 5 |
| CHRU Lille | Polyvalent | 0-10 | 10 |
| CHRU Nancy | Medical | 0-10 | 0 |
| CHU Grenoble | Medical | 0-10 | 5 |
